# Alternative lengthening of telomeres (ALT) influences survival in soft tissue sarcomas: a systematic review with meta-analysis

**DOI:** 10.1186/s12885-019-5424-8

**Published:** 2019-03-14

**Authors:** Rita T. Lawlor, Nicola Veronese, Antonio Pea, Alessia Nottegar, Lee Smith, Camilla Pilati, Jacopo Demurtas, Matteo Fassan, Liang Cheng, Claudio Luchini

**Affiliations:** 1 ARC-Net Research Center, University and Hospital Trust of Verona, Verona, Italy; 2 National Institute of Gastroenterology-Research Hospital, IRCCS “S. de Bellis”, Castellana Grotte, Bari, Italy; 3 National Research Council, Neuroscience Institute, Aging Branch, Padua, Italy; 4 Department of General and Pancreatic Surgery, The Pancreas Institute, University and Hospital Trust of Verona, Verona, Italy; 5 Department of Surgery, Section of Pathology, San Bortolo Hospital, Vicenza, Italy; 6 Cambridge Centre for Sport and Exercise Sciences, Anglia Ruskin University, Cambridge, UK; 7 Personalized Medicine, Pharmacogenomics, Therapeutic Optimization, Université Paris Descartes, Paris, France; 8 Primary Care Department, Azienda USL Toscana Sud Est, Grosseto, Italy; 9 Department of Pathology and Laboratory Medicine, Indiana University School of Medicine, Indianapolis, IN USA; 10 Department of Diagnostics and Public Health, Section of Pathology, University and Hospital Trust of Verona, Piazzale Scuro, 10, 37134 Verona, Italy

**Keywords:** ALT, Sarcoma, Mesenchymal, Prognosis, Survival, ATR, ATRX

## Abstract

**Background:**

Alternative lengthening of telomeres (ALT) is a telomerase-independent mechanism used by a broad range of neoplasms to maintain telomere length, permitting uncontrolled replication during their progression. ALT has been described in different types of sarcoma, but a comprehensive analysis of its clinical significance is still lacking. Therefore, we provide here the first meta-analysis on this topic.

**Methods:**

We searched SCOPUS and PubMed through July 2018 to identify all studies that investigated the prognostic role of ALT in sarcomas. We considered the risk of death (risk ratio, RR) calculated as the number of death vs. total participants during follow-up in ALT+ versus ALT- patients as the primary outcome. The secondary outcome was the hazard ratio (HR), adjusted for the maximum number of covariates available, using ALT- patients as reference.

**Results:**

Eight articles comprising a total of 551 patients with sarcomas (226 ALT+ and 325 ALT-) were selected. The ALT+ group showed a higher mitotic count and a higher tumor grade compared with the ALT- group (*p* < 0.01). Furthermore, we demonstrate a strong impact of ALT on survival. In fact, ALT+ patients showed a statistically significant higher risk of death than ALT- patients, when also considering data from multivariate analyses (RR = 1.50; 95% CI: 1.15–1.96; *p* = 0.003; HR = 2.02; 95% CI: 1.22–3.38; *p* = 0.007).

**Conclusions:**

Our results indicate that ALT is associated with an increased risk of death in patients with sarcoma. In these neoplasms, ALT should be taken into account for a precise prognostic stratification and design of potential therapeutic strategies.

**Electronic supplementary material:**

The online version of this article (10.1186/s12885-019-5424-8) contains supplementary material, which is available to authorized users.

## Background

In somatic cellular compartment, each round of cell division is accompanied by telomere shortening [1, 2]. This process is physiologically due to the inability of somatic cells to polymerize all DNA during replication. In cancer, this same process is overcome by the cancer cells in order to initiate and support cancer growth [3]. In fact, a hallmark of cancer is its unlimited replicative capacity, which can be achieved through the activation of so-called telomere maintenance mechanisms [3]. In the majority of cancers, telomere shortening is counterbalanced by the enzymatic activity of telomerase, a DNA-polymerase that provides de novo synthesis of telomeric DNA by reverse transcription of a RNA template [4–6]. However, a subset of neoplasms, mainly represented by sarcomas and gliomas, lacks telomerase and therefore maintains telomere length through a different telomerase-independent process, called alternative lengthening of telomeres (ALT) [7]. This mechanism concerns the synthesis of new telomeric DNA on the basis of a DNA template, where the telomere can either use itself, a telomere on a sister chromatid or even another chromosomes as the copy template [8, 9]. For this reason, ALT cells are characterized by long and heterogeneous telomeres and comprise sub-nuclear structures, such as APB (ALT-associated promyelocytic leukemia bodies) that contain telomeric DNA, and telomere-specific binding proteins, called TRF (terminal restriction fragments) [10].

Sarcomas represent a rare and heterogeneous group of mesenchymal neoplasms, with different clinical manifestations and biological behavior. A recently proposed classification of these tumors distinguishes two classes of sarcomas, one with simple karyotypes involving specific genetic alterations, and one with complex and unbalanced karyotypes, usually involving non-specific genetic abnormalities, including copy number alterations [11]. Despite such a complex molecular landscape, a relatively common cytogenetic anomaly of sarcomas, represented by ALT, has been demonstrated in both classes [12]. However, a comprehensive analysis of the clinical significance of ALT for this kind of neoplasms is still lacking. To this end, we assessed the prognostic role of ALT in sarcomas by performing the first systematic review with meta-analysis on this topic.

## Methods

The systematic review adhered to the MOOSE guidelines [13] and the PRISMA statement [14], based on a preset protocol (Additional file 1 Table S1).

### Inclusion and exclusion criteria

Studies were considered eligible if they met the following inclusion criteria: 1) involved a prospective cohort or retrospective study design; 2) investigated sarcomas either in general or a specific sarcoma subtype; 3) included a genetically-demonstrated presence of ALT; 4) included a comparison of prognostic factors among patients with ALT (ALT+) vs. without ALT (ALT-); 5) included data on mortality both overall and cancer-specific; and 6) the publication appeared in a peer-reviewed journal in the English language. Exclusion criteria were: 1) no mention of sarcoma of any subtype, 2) no mention of prognostic parameters in the title/abstract; 3) only indirect analysis of ALT associated alterations (e.g. mutational status of ALT-associated genes); and 4) in vitro or animal studies. In the case of doubled cohorts, the largest one was selected.

### Data sources and literature search strategy

Two investigators (RTL, CL) independently searched the PubMed, SCOPUS, EmBase and ISI databases up to July 17th 2018. The search terms included combinations of the following keywords: (“alternative lengthening of telomeres”) AND (mortality OR mortalities OR fatality OR fatalities OR death* OR survival OR prognosis OR “hazard ratio” OR HR OR “relative risk” OR RR). All references from all selected articles were also considered.

### Study selection

Following the database searches outlined above, duplicates were removed and two reviewers (RTL, AP) independently reviewed both the titles and abstracts of all the potentially eligible articles. The two authors applied the eligibility criteria and considered the full texts. A final list of articles was drawn up by consensus with a third independent author (NV).

### Data extraction

Two authors were involved in extracting the data into a preset Excel database: one (AP) extracted the data from the selected articles; the other (CL) independently validated the data. For each article, the following information was extracted: authors; year of publication; country; type of sarcoma; methods of ALT assessment; other ALT-associated parameters that were included in the studiesd; number of patients, together with their gender and age, data on mitotic index, tumor grade, number of adjustments in the case of multivariable analysis, mean or median follow-up.

### Outcomes

The primary outcome was represented by the number of deaths after treatment during follow-up in ALT+ versus ALT- patients (considering all-cause mortality and cancer-specific mortality together). The secondary outcome was represented by the hazard ratio (HR), adjusted for the maximum number of covariates available, using ALT- patients as the reference. Five-year follow-up data were extracted, where available; otherwise, the maximum reported follow-up was considered.

### Study quality assessment

The Newcastle-Ottawa Scale (NOS) was used to evaluate the quality of the studies [15]. This provides an assessment of the methodological quality of observational studies [16]. The studies were evaluated on the basis of eight elements across three key areas: selection of participants, comparability of participants, and outcomes. One author (CL) designed and completed the NOS and each study was attributed an overall score for methodological quality of up to nine points.

### Data synthesis and statistical analysis

All analyses were conducted using Comprehensive Meta-Analysis v2 software (Biostat; Englewood, NJ, USA). In our primary and secondary analyses, pooled RR and HR with corresponding 95% confidence intervals (CI) were calculated for mortality, comparing ALT+ with ALT- cases using DerSimonian-Laird random-effects models [17].

Heterogeneity across studies was assessed with I^2^ metric and chi square statistics [18]. A meta-regression analysis was performed in cases of high heterogeneity (i.e., I^2^ ≥ 50%) [19]. Eventual outlier studies were removed in a sensitivity analysis. Finally, we investigated publication bias by visual inspection of funnel plots and with the Egger bias test [20].

## Results

### Search results

The literature search identified a total of 371 unduplicated articles, 345 of which were excluded after review of their titles/abstracts, resulting in 26 articles eligible for full text review. After application of our inclusion criteria, eight articles, with a total of nine cohorts, were included in the meta-analysis (Additional file 2: Figure S1) [21–28].

### Characteristics of studies and patients

As shown in Additional file 3: Table S2, the 8 articles selected for the meta-analysis included a total of 9 different cohorts of patients comprising 551 patients with sarcomas (226 ALT+ and 325 ALT-) with a median follow-up period of 52.2 months (range: 28–92).

The ALT+ group had a statistically significant higher mitotic count compared with the ALT- group (*p* = 0.0078, Fisher’s exact test), in addition to a statistically significant higher tumor grade (*p* < 0.0001). The ALT+ group comprised a higher proportion of women than ALT- (77% vs. 60%); ALT+ patients were also older than ALT- (54.9 vs 50.9 years old), but these differences were not statistically significant.

The median NOS score indicated the overall good quality of the studies (median, 7 points; range, 6–9), with no manuscripts displaying a high risk of bias (Additional file 3: Table S2 and Additional file 4: Table S3).

Two studies investigated the prognostic role of ALT in liposarcoma [21, 24] and two others in leiomyosarcoma [25, 27]; the remaining five cohorts investigated other types of sarcoma (Additional file 3: Table S2). ALT status was assessed by the evaluating APB in six cohorts (TRF was also evaluated in two of these) and by fluorescent in situ hybridization (FISH) in the remaining three (Additional file 3: Table S2).

### Risk ratio for survival

Table 1 shows the risk ratios estimated by ALT status.

**Table 1:** Risk Ratio and Hazard Ratio for survival on the basis of ALT status

Parameter	N Cohorts	Risk Ratio (95% CI)	*p* Value	Heterogeneity (I^2^%); tau^2^	Egger Test (*p* value)
Survival	8	1.50 (1.15–1.96)	0.003	53%; 0.07	1.49 (0.17)
Parameter	N Cohorts	Hazard Ratio (95% CI)	*p* Value	Heterogeneity (I^2^); tau^2^	Egger Test (*p* value)
Survival	6	2.02 (1.22–3.38)	0.007	49.8%; 0.19	1.40 (0.57)

**Fig. 1:**
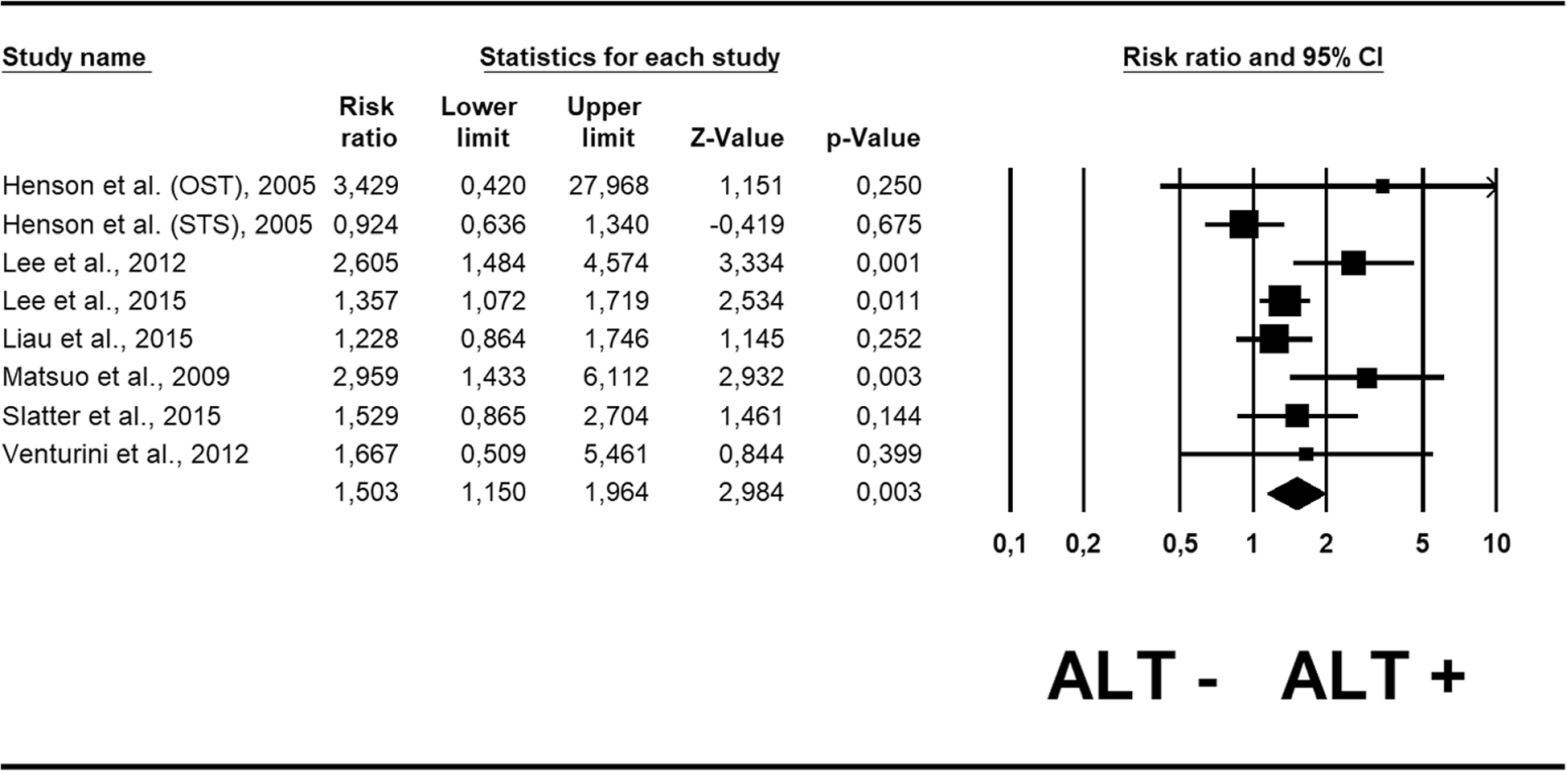
Forest plot of risk ratio for mortality in sarcoma patients

Pooling the results from the 8 cohorts reporting RR [24–30], we found that ALT+ patients had a statistically significant higher risk of death than ALT- patients (RR = 1.50; 95% CI: 1.15–1.96; *p* = 0.003) (Fig. 1). The one unique outlier-study, that investigated a cohort of osteosarcoma patients [22], was removed in the sensitivity analysis, and the RR was re-calculated. It maintained the statistical significance, showing a similar value (RR = 1.48; 95% CI: 1.13–1.95; *p* = 0.001). No publication bias emerged for this outcome (Egger’s test = 1.49 ± 0.95; *p* = 0.17), as also shown by the funnel plot (Additional file 5: Figure S2A).

### Hazard ratio for survival

Table 1 shows also the HR estimated by ALT status. In the case of adjusted analysis, the median number of adjustments was 2.25 (range 1–3) (Additional file 6: Table S4). Pooling the results from the 6 cohorts reporting HR adjusted for potential confounders [21, 22, 25, 26, 28], we found that ALT+ doubled the risk of death, with a statistically significant value (HR = 2.02; 95% CI: 1.22–3.38; *p* = 0.07). Similar to the sensitivity analysis conducted for RR, we removed the same unique outlier study on osteosarcoma [22]: the recalculated HR maintained statistical significance, showing a slightly higher value (HR = 2.08; 95 CI: 1.18–3.66; *p* = 0.01). No publication bias emerged either for this outcome (Egger’s test = 1.40 ± 2.26; *p* = 0.57), as also shown by the funnel plot (Additional file 5: Figure S2B).

### Meta-regression analyses

Only risk ratio of survival demonstrated high heterogeneity (I^2^ = 53%). We therefore performed a meta-regression analysis for this outcome, searching for potential moderators of the heterogeneity, and found that it may be partly explained by the presence of different sarcoma histotypes. In fact, a sub-analysis considering only leiomyosarcoma showed a drastic reduction of heterogeneity (I^2^ = 0%), while it remained high (I^2^ = 64%) when considering the remaining studies altogether (all studies analyzing different sarcoma types).

## Discussion

In this study, we analyzed 9 different cohorts involving 551 patients with sarcomas. Of these, 226 were ALT+ while 325 were ALT-. Our findings indicate that activation of the ALT mechanism is associated with an increased risk of mortality in this type of tumor. Noticeably, this association is reinforced when considering HR adjusted for potential confounders. Furthermore, no risk of publication bias was documented, highlighting the robustness of the findings. Based on our results, the ALT mechanism appears to have a strong clinical impact in sarcomas (doubled risk of mortality, HR = 2.02). Given that this meta-analysis pooled together the different risks of mortality of several sarcoma types, the high statistical significant values of ALT further highlight its widely applicable prognostic value.

**Fig. 2:**
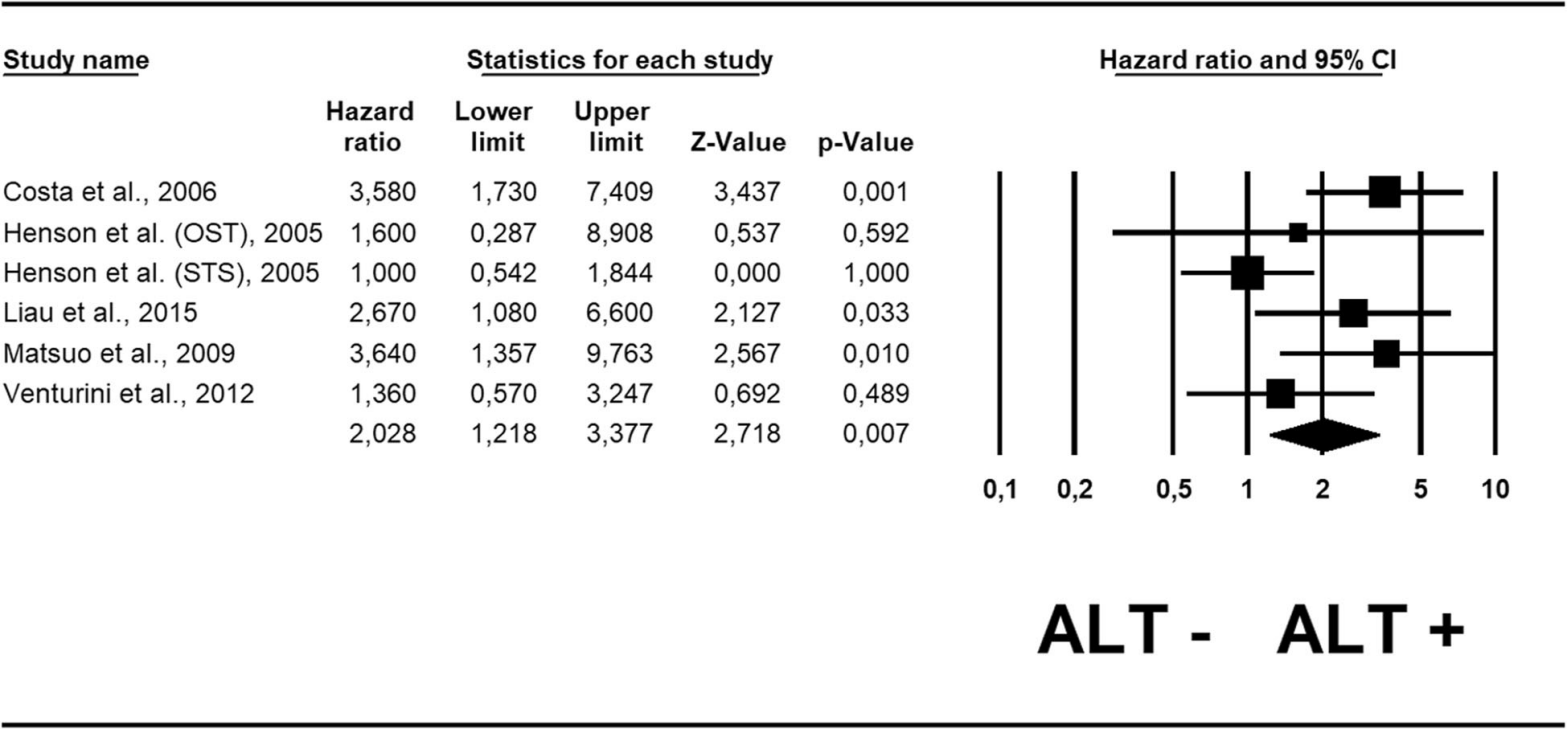
Forest plot of hazard ratio for mortality in sarcoma patients

ALT has been demonstrated to be an important biological process in different cancer types [7], and in particular neoplasms it has a strong prognostic significance. For example, in neuroendocrine tumors of the pancreas (PanNET), the activation of this mechanism is associated with an increased risk of mortality and of distant metastasization, even in low-grade neoplasms [29–31]. Interestingly, in pediatric malignant brain tumors, the presence of ALT is associated with a better prognosis, above all in cases of concomitant *TP53* mutation [32]. The present study demonstrates that ALT plays an important prognostic role in sarcoma, as it is associated with an increased risk of death. Given that ALT was associated with a higher mitotic count and a higher tumor grade in our analysis, the fact that it maintained its prognostic significance also when using data from multivariable analysis (adjusted among others also for tumor grade, Fig. 2**,** Additional file 6: Table S4) further highlights its clinical importance. The one exception to the poor prognostic value of ALT in the heterogeneous group of sarcomas appears to be osteosarcoma. Although there are few data upon which to draw definitive conclusions, Sanders and colleagues suggested that patients with ALT+ osteosarcoma may have a diminished risk of tumor recurrence [33]. While our meta-analysis demonstrated reliable results regarding the poor prognostic value of ALT in soft tissue sarcoma, further studies are needed to advance current knowledge about concerning ALT and prognosis.

The activation of the ALT mechanism has been also shown to closely correlate with the mutational status of the chromatin remodeling genes *DAXX* (death domain-associated protein) and *ATRX* (α-thalassemia/mental retardation X-linked) in different malignancies, including PanNET and pediatric glioblastoma [30, 31, 34–38]. *DAXX* and *ATRX* may be also mutated in different types of sarcomas [12, 27, 39, 40]. These genes encode nuclear proteins essential in regulating the deposition of histone variant H3.3 during the assembly of pericentromeric and telomeric chromatin [41]. *DAXX*/*ATRX* mutations may be studied not only with molecular analysis but also with immunohistochemistry, representing a widely-accepted surrogate of their mutational status, that shows loss of the encoded proteins associated with their mutations [30, 31]. In general, mutations affecting *DAXX* are more common in PanNET [30, 31, 42], whereas those affecting *ATRX* are markedly prominent in sarcomas [39, 40]. While in PanNET DAXX/ATRX immunohistochemical loss is absolutely associated with ALT+, the situation appears more complex in sarcomas. In fact, only about 50% of ALT+ leiomyosarcomas and pleomorphic liposarcomas are also ATRX deficient [24, 25]; at the same time ALT+ appears to be highly correlated with ATRX loss in angiosarcomas and dedifferentiated liposarcomas [24, 43]. Therefore, in the heterogeneous tumor group of sarcomas, other biological (both genetic and epigenetic) changes (still largely unknown) may concur in activating ALT and determining the so-called “ALT phenotype”. In the complex background of ALT-*DAXX*/*ATRX* interactions, our findings concerning the reliability of ALT as a strong prognostic moderator appear of greater importance.

The evaluation of ALT also has potential implications for targeted therapeutic strategies. Recent evidence has indicated that the inhibition of the protein kinase ATR (ataxia telangiectasia and Rad3-related protein), a crucial regulator of DNA recombination, can disrupt ALT by triggering chromosome fragmentation and apoptosis in ALT cells [43]. Cell death induced by ATR inhibition is highly selective for ALT+ cells, suggesting ATR inhibitors as potentially useful in the treatment of ALT+ cancers [9, 44–46]. Notably, some ATR inhibitors have been already tested in vivo for cancer treatment with encouraging results [47–49]. As we have highlighted that the activation of this mechanism has a poor prognostic impact in sarcomas, this potential therapeutic approach could also be explored in this tumor category. The integration of ALT status in pathology reports may therefore provide useful prognostic and therapeutic information for sarcomas as part of a next-generation histopathological approach [50].

This analysis presents results based on the review of a number of studies that do, however, have some limitations worth considering. The first concerns the overall sample size but this is, in part, due to the rarity of the tumor type. That said, the presence of different sarcoma types in the analysis adds some level of reliability. Additionally, data regarding possible co-morbidities such as cardiovascular diseases were not reported in the studies, although these are known to play an important role in the prognosis of these cancer patients. Finally, the different biological behavior of ALT in osteosarcoma [33] compared to all other sarcoma sub-types requires additional data on survival to further explore this particular aspect.

## Conclusions

In conclusion, our results indicate that ALT+ is associated with an increased risk of death in patients with soft tissue sarcoma, even after adjusting for potential confounders. As this mechanism is present in a significant proportion of this tumor type, its determination appears of primary importance for a correct prognostic stratification of such patients and for designing future therapeutic strategies.

## Additional files

Additional file 1: **Table S1.** PRISMA checklist. The PRISMA checklist for this systematic-review and meta-analysis is here provided. (DOC 64 kb)

Additional file 2: **Figure S1.** PRISMA checklist for this meta-analysis. The PRISMA figure representing all the steps for selecting/screening papers for this systematic-review and meta-analysis is here provided. (DOCX 45 kb)

Additional file 3: **Table S2.** Characteristics of the studies according to Alternative Lengthening of Telomeres (ALT). This summarizing table shows the different features of all studies included in this systematic review and meta-analysis. (DOCX 29 kb)

Additional file 4: **Table S3.** Methodological quality of cohort studies included in the meta-analysis. This summarizing table shows the method of quality assessment of all the studies included in this systematic review and meta-analysis, using the Newcastle-Ottawa scale. (DOCX 16 kb)

Additional file 5: **Figure S2.** Funnel plots. Funnel plots for risk ratio (A) and hazard ratio (B) for this systematic review and meta-analysis are here provided. (TIF 1991 kb)

Additional file 6: **Table S4.** Type and number of adjustments (in addiction of ALT status) for each study. This summarizing table shows the different adjustments of all studies, which have investigated ALT status with multivariate analysis. (DOCX 20 kb)

## Abbreviations

ALT: Alternative lengthening of telomeres
APB: ALT-associated promyelocytic leukemia bodies
ATR: Ataxia telangiectasia and Rad3-related protein
ATRX: α-thalassemia/mental retardation X-linked
CI: Confidence intervals
DAXX: Death domain-associated protein
FISH: Fluorescent in situ hybridization
HR: Hazard ratio
NOS: Newcaste-Ottawa Scale
PanNET: neuroendocrine tumors of the pancreas
RR: Risk ratio
TP53: Tumor protein 53
TRF: Terminal restriction fragments
