# Supplementary material for: Alternative lengthening of telomeres (ALT) influences survival in soft tissue sarcomas: a systematic review with meta-analysis
Source: BMC Cancer. 2019 Mar 14;19:232. doi: 10.1186/s12885-019-5424-8 (PMC6419345; doi:10.1186/s12885-019-5424-8)
Supplement: Supplementary file 2 — Figure S1. PRISMA checklist for this meta-analysis. The PRISMA figure representing all the steps for selecting/screening papers for this systematic-review and meta-analysis is here provided. (DOCX 45 kb) [file 12885_2019_5424_MOESM2_ESM.docx]

**Supplementary Figure 1. PRISMA checklist for this meta-analysis.**

Records after duplicates removed
(n = 371)

Additional records identified through other sources
(n = 1)

Records identified through database searching
(n = 566)

## Identification

## Screening

Records excluded
(n = 345)

Records screened
(n = 371)

Full-text articles excluded, with reasons
(n = 18)

*Doubled cohort (n=5)*

*No survival data (n=8)*

*Reviews (n=2)*

*No control group (n=3)*

## Eligibility

Full-text articles assessed for eligibility
(n = 26)

Studies included in quantitative synthesis (meta-analysis)
(n = 8, for 9 cohorts)

## Included
