# Supplementary material for: Alternative lengthening of telomeres (ALT) influences survival in soft tissue sarcomas: a systematic review with meta-analysis
Source: BMC Cancer. 2019 Mar 14;19:232. doi: 10.1186/s12885-019-5424-8 (PMC6419345; doi:10.1186/s12885-019-5424-8)
Supplement: Supplementary file 3 — Table S2. Characteristics of the studies according to Alternative Lengthening of Telomeres (ALT). This summarizing table shows the different features of all studies included in this systematic review and meta-analysis. (DOCX 29 kb) [file 12885_2019_5424_MOESM3_ESM.docx]

**Supplementary Table 2.** Characteristics of the studies according to Alternative Lengthening of Telomeres (ALT)

|  | | | | | **ALT+** | | | | | **ALT-** | | | | |  | | |
| --- | --- | --- | --- | --- | --- | --- | --- | --- | --- | --- | --- | --- | --- | --- | --- | --- | --- |
| **Study**  **Author, Year (Country)** | **Type of sarcoma** | **Exclusion**  **criteria** | **Methods of ALT assessment** | **Other analysis about telomere-associated variables** | **Number of participants** | **N. of females**  **(%)** | **Mean Age ± SD** | **Mitotic index** | **Tumor Grading** | **Number of participants** | **N. of females**  **(%)** | **Mean Age ± SD** | **Mitotic index** | **Tumor Grading** | **Number of adjustments** | **NOS** | **Mean**  **Follow-up period**  **(months)** |
| Costa, 2006  (Italy) | Liposarcoma | NA | APB | Telomerase activity | 36 | NA | NA | NA | Low: 41.7%; High: 58.3% | 103 | NA | NA | NA | Low: 56.3%; High: 43.7% | 3 | 9 | 92 |
| Henson, 2005 (USA) | Soft tissue sarcoma | Lack of follow-up information | APB, TRF | Telomerase activity, Telomere length | 33 | NA | NA | NA | NA | 32 | NA | NA | NA | NA | 0 | 7 | >60 |
| Henson, 2005 (Australia) | Osteosarcoma | Lack of follow-up information | APB, TRF | Telomerase activity, Telomere length | 21 | NA | NA | NA | NA | 18 | NA | NA | NA | NA | 0 | 6 | 28 |
| Lee, 2012 (Republic of Korea) * | Uterine sarcoma and carcinosarcoma | Lack of follow-up information | APB, C-circle assay | Telomerase activity | 19 | 100% | <50 y: 36.8%; ≥50 y: 63.2% | Low: 5.3%; High: 94.7% | Low: 25%; High: 75% | 22 | 100% | <50 y: 54.5%; ≥50 y: 45.5% | Low: 45.5%; High: 54.5% | Low: 76.9%; High: 23.1% | 0 | 8 | >60 |
| Lee, 2015 (Taiwan) | Dedifferentiated liposarcoma | NA | FISH | IHC for ATRX and DAXX | 14 | 43% | Median: 69 y | NA | Low: 14%; High: 86% | 32 | 37% | Median 61 y | NA | Low: 75%; High: 25% | 0 | 8 | >60 |
| Liau, 2015 (Taiwan) | Leiomyosarcoma | DPC | FISH | IHC for ATRX and DAXX, *TERT* mutation | 51 | 86% | Mean; 52.5 y | Low: 11.9%; High: 88.1% | Low: 40%; High: 60% | 35 | 80% | Mean: 57.6 y | Low: 29.0%; High: 71.0% | Low: 68%; High: 32% | 3 | 7 | 37 |
| Matsuo, 2009 (Japan) | Malignant Fibrous Histiocytomas | NCT | FISH | Telomerase activity, Telomere length | 14 | NA | NA | NA | NA | 29 | NA | NA | NA | NA | 2 | 7 | NA |
| Slatter, 2015 (New Zealand) ** | Uterine Leiomyosarcoma | Death to other cause, no data at follow-up | APB | IHC for ATRX and DAXX | 17 | 100% | Mean: 50.5 y | Low: 17.7%; High: 82.3% | NA | 26 | 100% | Mean: 49.1 y | Low: 38.5%; High: 61.5% | NA | 0 | 7 | 35 |
| Venturini, 2012 (Italy) | Peripheral nerve sheath tumor | No data at follow-up | APB, TRF | Telomerase activity | 21 | NA | NA | NA | NA | 28 | NA | NA | NA | NA | 1 | 7 | 46 |
| **Total**  **Studies**  **(weighted values*)* ***** | **2 Liposarcoma; 1 Osteosarcoma; 2 Leiomiosarcoma; 4: others** | **-** | **PM: 6 APB, 3 FISH** | **6: telomerase activity** | **226** | **77%** | **54.9 y** | **Low: 11.3%; High: 88.7%** | **Low: 35%; High: 65%** | **325** | **60%** | **50.9 y** | **Low: 36.1%; High: 63.9%** | **Low: 60.9%; High: 39.1%** | **Range: 0-3** | **Median: 7** | **Mean: 52.2** |

Notes: *for this study, data on tumor grading were extractable only for endometrial stromal sarcomas; **Patients of this study were from New Zealand or China; ***for the final summary of percentages of females vs. male, studies on female genital tumors (100% females) have been excluded.

Abbreviations: APB: ALT-associated promyelocytic leukemia bodies; TRF: terminal restriction fragments; FISH: fluorescent in situ hybridization; IHC: immunohistochemistry; ATRX: α-thalassemia/mental retardation syndrome X-linked protein; DAXX: domain associated protein 6; *TERT*: telomerase reverse transcriptase gene; DPC: death for post-operative complications; NCT: Neo-adjuvant chemotherapy; PM: primary method of ALT assessment.
