# Supplementary material for: Alternative lengthening of telomeres (ALT) influences survival in soft tissue sarcomas: a systematic review with meta-analysis
Source: BMC Cancer. 2019 Mar 14;19:232. doi: 10.1186/s12885-019-5424-8 (PMC6419345; doi:10.1186/s12885-019-5424-8)
Supplement: Supplementary file 4 — Table S3. Methodological quality of cohort studies included in the meta-analysis. This summarizing table shows the method of quality assessment of all the studies included in this systematic review and meta-analysis, using the Newcastle-Ottawa scale. (DOCX 16 kb) [file 12885_2019_5424_MOESM4_ESM.docx]

**Supplementary Table 4.Type and number of adjustments (in addiction of ALT status) for each study.**

| **First author,**  **publication year** | **Adjustments** | **Maximum number of adjustments** |
| --- | --- | --- |
| Costa, 2006 | Tumor location, G, histology | 3 |
| Henson, 2005 (STS) | - | 0 |
| Henson, 2005 (OST) | - | 0 |
| Lee, 2012 | - | 0 |
| Lee, 2015 | - | 0 |
| Liau, 2015 | Tumor site, tumor differentiation, G | 3 |
| Matsuo, 2009 | Tumor size, Telomerase activity | 2 |
| Slatter, 2015 | - | 0 |
| Venturini, 2012 | Margin status | 1 |

Abbreviations: G: tumor grade; STS: soft tissue sarcoma cohort; OST: osteosarcoma cohort.
