# Supplementary figures and images for: Alternative lengthening of telomeres (ALT) influences survival in soft tissue sarcomas: a systematic review with meta-analysis
Source: BMC Cancer. 2019 Mar 14;19:232. doi: 10.1186/s12885-019-5424-8 (PMC6419345; doi:10.1186/s12885-019-5424-8)

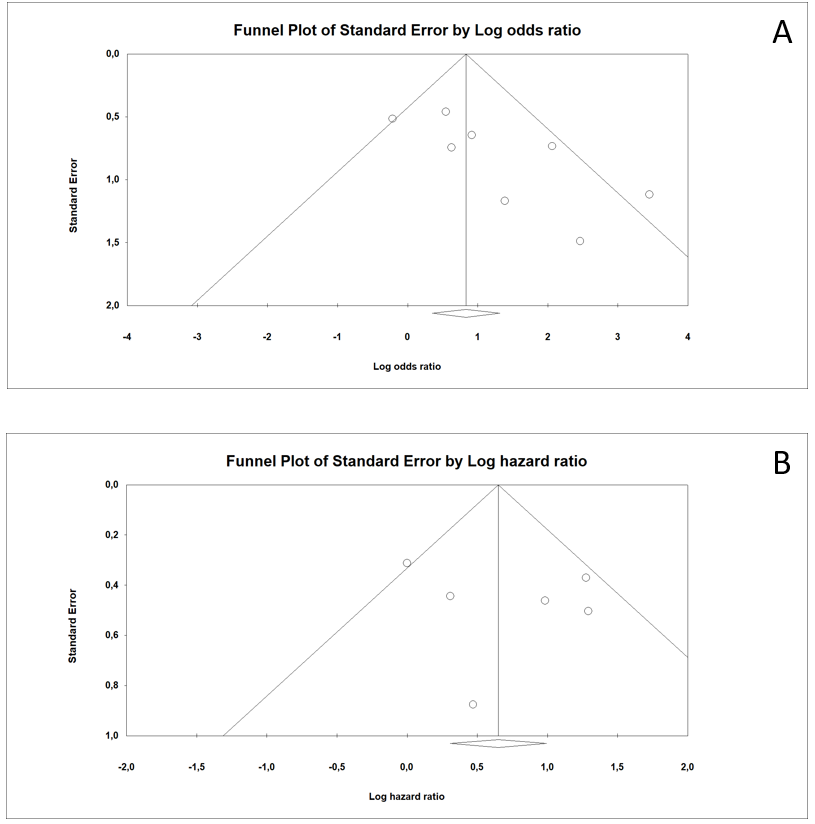

Supplement: Supplementary file 5 — Figure S2. Funnel plots. Funnel plots for risk ratio (A) and hazard ratio (B) for this systematic review and meta-analysis are here provided. (TIF 1991 kb) [file 12885_2019_5424_MOESM5_ESM.tif]
