# Supplementary material for: Alternative lengthening of telomeres (ALT) influences survival in soft tissue sarcomas: a systematic review with meta-analysis
Source: BMC Cancer. 2019 Mar 14;19:232. doi: 10.1186/s12885-019-5424-8 (PMC6419345; doi:10.1186/s12885-019-5424-8)
Supplement: Supplementary file 6 — Table S4. Type and number of adjustments (in addiction of ALT status) for each study. This summarizing table shows the different adjustments of all studies, which have investigated ALT status with multivariate analysis. (DOCX 20 kb) [file 12885_2019_5424_MOESM6_ESM.docx]

**Supplementary Table 3.** Methodological quality of cohort studies included in the meta-analysis*

| **First author,**  **publication year** | **Representativeness**  **of the exposed cohort** | **Selection of the unexposed**  **cohort** | **Ascertainment**  **of exposure^†^** | **Outcome of interest**  **not present**  **at start of study^††^** | **Control for**  **important factor or additional factor^†††^** | **Assessment of outcome** | **Follow-up**  **long enough for outcomes**  **to occur^††††^** | **Adequacy of**  **follow-up**  **of cohorts** | **Total quality**  **scores** |
| --- | --- | --- | --- | --- | --- | --- | --- | --- | --- |
| Costa, 2006 | * | * | * | * | ** | * | * | * | 9 |
| Henson (STS), 2005 | * | * | * | - | * | * | * | * | 7 |
| Henson (OST), 2005 | * | * | * | - | * | * | - | * | 6 |
| Lee, 2012 | * | * | * | * | * | * | * | * | 8 |
| Lee, 2015 | * | * | * | * | * | * | * | * | 8 |
| Liau, 2015 | * | * | * | - | ** | * | - | * | 7 |
| Matsuo, 2009 | * | * | * | - | ** | * | - | * | 7 |
| Slatter, 2015 | * | * | * | * | * | * | - | * | 7 |
| Venturini, 2012 | * | * | * | * | * | * | - | * | 7 |
|  |  |  |  |  |  |  |  |  |  |

Original studies were analyzed in the quality assessment.

* A study could be awarded a maximum of one star for each item except for the item Control for important factor or additional factor. The definition/explanation of each column of the Newcastle-Ottawa Scale is available at <http://www.ohri.ca/programs/clinical_epidemiology/oxford.htm>.

^†^ For this index, one star was given if in Method section ALT assessment was clearly defined (e.g.: defining ALT-associated promyelocytic leukemia bodies)

^††^ Being outcome of interest mortality, we took as outcome of interest for assessment of quality if the cancer-specific mortality or the risk of recurrence was assessed.

^†††^ A maximum of 2 stars could be awarded for this item. Studies that controlled their survival analyses for at least two confounders received one star, whereas studies that investigated also telomerase activity or telomere-associated variables, an additional star.

**^††††^** A cohort study with a mean/median follow-up time ≥5 y (60 months) takes one star.

Abbreviations: STS: soft tissue sarcoma cohort; OST: osteosarcoma cohort;
